# Heterotrophic Soil Microbes at Work: Short-Term Responses to Differentiated Fertilization Inputs

**DOI:** 10.3390/biology15010041

**Published:** 2025-12-26

**Authors:** Florin Aonofriesei, Alina Giorgiana Brotea (Andriescu), Enuță Simion

**Affiliations:** Department of Natural Sciences, Faculty of Natural and Agricultural Sciences, Ovidius University of Constanta, University Street, 900470 Constanța, Romania; simionenuta2003@yahoo.com

**Keywords:** fertilizers, bacteria, rhizosphere, dehydrogenase activity

## Abstract

The interaction between organic and inorganic nutrients, bacterial communities, and soil fertility has been well documented over time. Conventional agricultural systems heavily utilize both inorganic and organic fertilizers, each exerting distinct effects on soil microbial dynamics and plant growth. The objective of our experiments was to identify the most effective fertilization strategy for improving the biological quality of a microbiologically impoverished and low-productivity soil. To this end, four fertilization strategies were evaluated: (i) organic fertilizers characterized by a high content of organic carbon (Fertil 4-5-7—variant 1); (ii) organic fertilizers with 12% organic nitrogen from proteins (Bio Ostara N—variant 2) (iii) combined inorganic–organic fertilizers (P35 Bio—variant 3) and (iv) mineral (inorganic) fertilizers (BioAktiv—variant V4). This study aimed to assess the short-term effects of fertilizers with varying chemical compositions on the density of cultivable heterotrophic bacteria and their associated dehydrogenase (DH) activity in a petrocalcic chernozem soil containing pedogenic carbonates. Soil sampling was conducted according to a randomized block design, comprising four replicates per treatment (control plus four fertilizer types). The enumeration of cultivable bacteria was performed using Nutrient Agar and A2R Agar media, whereas dehydrogenase activity (DHA) was quantified based on the reduction of 2,3,5-triphenyl-2H-tetrazolium chloride (TTC) to 1,3,5-triphenyl-tetrazolium formazan (TPF) by bacterial dehydrogenase enzymes. Marked differences were observed in both parameters between the plots amended with inorganic fertilizers and those treated with organic fertilizers, as well as among the organic fertilizer treatments of varying composition. The most pronounced increases in both bacterial density and dehydrogenase activity (DHA) were recorded in the plots receiving the fertilizer with a high organic nitrogen content. In this treatment, the maximum bacterial population density reached 6.25 log_10_ CFU g^−1^ dry soil after approximately two months (May), followed by a significant decline starting in July. In contrast, DHA exhibited a more rapid response, reaching its peak in April (42.75 µg TPF g^−1^ soil), indicating an earlier DHA activation of microbial metabolism. This temporal lag between the two parameters suggests that enzymatic activity responded more swiftly to the nutrient inputs than did microbial biomass proliferation. For the other two organic fertilizer variants, bacterial population dynamics were broadly similar, with peak densities recorded in June, ranging from 5.98 log_10_ CFU g^−1^ soil (V3) to 6.03 log_10_ CFU g^−1^ soil (V1). A comparable trend was observed in DHA: in V3, maximum DHA was attained in June (30 µg TPF g^−1^ soil), after which it remained relatively stable, whereas in V1, it peaked in June (24.05 µg TPF g^−1^ soil) and subsequently declined slightly toward the end of the experimental period. Overall, the temporal dynamics of bacterial density and DHA demonstrated a strong dependence on the quality and biodegradability of the organic matter supplied by each fertilizer. Both parameters were consistently lower under inorganic fertilization compared with organic treatments, suggesting that the observed increases in microbial density and activity were primarily mediated by the enhanced availability of organic substrates. The relationship between the density of culturable heterotrophic bacteria and dehydrogenase (DH) activity was strongly positive (r = 0.79), indicating a close functional linkage between bacterial density and oxidative enzyme activity. This connection suggests that the culturable fraction of the heterotrophic microbial community plays a key role in the early stages of organic matter mineralization derived from the applied fertilizers, particularly in the decomposition of easily degradable substrates.

## Simple Summary

This study compared the effects of inorganic and organic fertilizers with different compositions on soil bacterial density and dehydrogenase activity (DHA), two key indicators of soil microbial activity. Results showed that organic fertilizers promoted higher microbial density and enzymatic activity than inorganic fertilizers. The strongest response occurred in the treatment containing a high proportion of organic nitrogen, where bacterial density reached 6.25 log_10_ CFU g^−1^ soil in May, followed by a decline in July. DHA, however, responded more rapidly, peaking earlier in April at 42.75 µg TPF g^−1^ soil, suggesting that enzymatic activity increased before microbial density increase. In the other organic fertilizer treatments, both parameters peaked in June, though at lower levels, and remained relatively stable or decreased slightly toward the end of the experiment. The results indicate that the quality and biodegradability of organic matter strongly influence microbial growth and metabolic activity. Overall, organic fertilization enhanced soil biological functioning compared with inorganic fertilization, primarily through the greater availability of carbon and nitrogen substrates that support microbial metabolism. These findings highlight the role of organic inputs in sustaining soil health and promoting long-term agricultural productivity.

## 1. Introduction

Essential nutrients such as nitrogen (N), phosphorus (P), and sulfur (S) are sequestered in soils as organic compounds, rendering them inaccessible to plants. Consequently, plants rely on microbial activity for the degradation and mineralization of these organic nutrients. Microbial biomass ultimately releases organic nutrients through cell lysis or protozoan predation [1]. Thus, microorganisms and their nutrient-transforming processes are critical for plant productivity and can be limiting factors in certain ecosystems [2]. The availability of nutrients for plant growth is significantly influenced by microbial community composition, physiological traits, and biomass, all of which are pivotal for nutrient cycling and plant nutrition [3,4,5]. The mineralized forms of N, P, and S, such as ammonium, nitrate, phosphate, and sulfate ions, can be readily assimilated by plants [6]. Soil microorganisms also play a vital role in recycling biogenic elements [7]. The soil microbiome constitutes the most diverse biotic community on Earth, accounting for a substantial portion of global biodiversity [8]. The rhizosphere is essential for processes and functions that facilitate nutrient recycling [9]. Understanding the interactions among plants, soil, and nutrients necessitates a comprehensive grasp of these dynamics [10]. Soil microorganisms perpetuate nutrient turnover through three mechanisms: (i) decomposition of organic macromolecules; (ii) incorporation and conversion of small molecules into microbial biomass; and (iii) the release of various metabolites upon microbial cell death, which interact with soil minerals. Through these dynamic processes characterized by the assimilation and mineralization of organic substrates, microorganisms function as a “microbial carbon pump” [11]. In soil, organic nitrogen is utilized following decomposition and transformation into mineral forms through microbial activity [3,12]. The spatial variability of soil nutrients is crucial to plant nutrition, as microbial communities can redistribute immobilized nutrients within heterogeneous soil matrices [2]. Microorganisms are integral to soil life and plant metabolism through their role in soil formation and development [10]. Dehydrogenases are oxidoreductase enzymes, and their activity serves as an indicator of microbial metabolic rates [13,14]. Most dehydrogenases facilitate hydrogen transfer to NAD^+^ and NADP^+^. They rely on the activity of various enzymes involved in metabolic processes (respiratory pathways, the tricarboxylic acid cycle, and nitrogen metabolism) [15]. Dehydrogenases are crucial for organic matter degradation by transferring hydrogen from organic substrates to inorganic acceptors [16]. Consequently, their activity in soil samples reflects microbial metabolic activity, which is tightly related to microbial biomass [14]. In contemporary agricultural systems, the supply of macronutrients is primarily accomplished through the application of mineral fertilizers. The reliance on mineral fertilizers can lead to numerous detrimental consequences, including eutrophication of aquatic ecosystems, greenhouse gas emissions, and degradation of soil health [17]. Consequently, current research aims to identify alternative strategies for the nutritional support of crops that significantly diminish the dependence on mineral fertilizers [18]. Transitioning away from mineral fertilizers involves both the utilization of organic byproducts and the intentional introduction of microorganisms that can decompose and mineralize these materials. Organic substrates can originate from diverse sources, predominantly as waste products; they can be easily composted and subsequently utilized as fertilizers [2]. Additionally, organic substrates have the advantage of being more stable within the soil environment, rendering them less susceptible to leaching and volatilization [19]. Several organic fertilizers are already employed in organic farming systems. However, the effectiveness of these bio-fertilizers remains contentious due to limited understanding of their impact on microbial communities and their efficacy in promoting the conversion of nutrients into plant-accessible mineral forms. Gaining insights into how microbial communities respond to different fertilizer treatments can assist in the development of effective management practices for sustainable agroecosystem production [20]. Considering these aspects, this study examines the relationship between the quality of organic and inorganic fertilizers and their effects on the density of culturable bacterial communities and dehydrogenase activity in petrocalcic chernozem soil with pedogenic carbonates. Although the culturable fraction constitutes only a minor component of the total soil microbiome, it is composed of species exhibiting rapid growth kinetics (*r*-strategists) [21,22,23]. *R*-strategist bacteria are characterized by rapid growth and reproduction under resource-rich and uncrowded conditions, displaying high specific growth rates and short life cycles. Conversely, *K*-strategist bacteria are adapted to stable, resource-limited, and densely populated environments, emphasizing survival, competitive ability, and metabolic efficiency rather than rapid proliferation [21,22,23]. These microorganisms possess a heightened capacity to decompose substantial quantities of labile organic substrates over short temporal scales, thereby accelerating the mineralization of organic matter and enhancing the bioavailability of essential macro- and micronutrients for plant uptake [24,25,26]. Soil microorganisms constitute a critical component of soil fertility and play a central role in agroecosystem functioning. Accordingly, the aim of this study was to identify the most effective fertilization strategy for improving the biological quality of a microbiologically depleted soil with low productive capacity. The experimental approach focused on enhancing the size and activity of soil microbial communities responsible for organic matter turnover, nutrient mineralization, and biogeochemical cycling, thereby increasing the availability of essential nutrients for plant uptake. Four fertilization strategies were evaluated: (i) organic fertilizers with a high organic carbon content; (ii) organic fertilizers rich in organically bound nitrogen (iii) integrated inorganic–organic fertilization and (iv) mineral (inorganic) fertilizers. The first two strategies two are predominantly associated with organic farming systems, where the enhancement of soil microbial biomass, functional activity, and ecological sustainability is a primary objective whereas the latter are widely applied in conventional agricultural systems, often with limited consideration of their effects on soil biological processes.

## 2. Materials and Methods

Sampling Site: This study was conducted on an experimental field affiliated with the Faculty of Natural and Agricultural Sciences at Ovidius University of Constanta. The experiments were implemented on rectangular plots, each measuring 40 m^2^. The experimental treatments consisted of the following: (i) Control group, which did not receive any fertilizers; (ii) V1—application of Fertil 4-5-7 (Organic Industries Infertosa, Valencia, Spain) at a rate of 500 kg/ha; (iii) V2—application of Bio Ostara N (Norofert SA, Bucharest, Romania) fertilizer at a rate of 250 kg/ha; (iv) V3—treatment with P35 Bio (Norofert SA, Bucharest, Romania) fertilizer at a rate of 200 kg/ha; and (v) V4—application of BioAktiv (BioAktiv International GmBH, Zeitz, Germany) fertilizer at a rate of 1.5 kg/ha. The fertilizers applied in this study were commercially available formulations, with their chemical composition and nutrient content specified by the manufacturers. Fertilizer application rates and methods were implemented in accordance with the manufacturers’ recommendations. Fertilizers were applied in November 2024 and February 2025, prior the start of experiments. Soil samples were collected between March and July 2025, during which the experimental plots were sown and cultivated with rapeseed, specifically the Umberto KWS variety. Fertilizer Composition: The fertilizers utilized in the experiments had the following compositions: (i) Fertil 4-5-7 (Organic Industries Infertosa, Spain): fertilizer based on organic substance of animal origin; organic matter 48%, total nitrogen 4.5%, total phosphorus 5%, total potassium 7%, calcium 8%, humic acids 23%; (ii) Bio Ostara N (Norofert SA, Bucharest, Romania): 100% blood meal with 12% organic nitrogen from crude protein with trace elements (Fe, Mn, Zn, Cu, B, Mo); (iii) P35 Bio (Norofert SA, Bucharest, Romania): organic fertilizer with a high phosphorus content of 28% in the form of phosphate rock; (iv) BioAktiv (BioAktiv International GmBH, Germany): magnesium sulfate 49%, K_2_SO_4_ 0.1%, CaSO_4_ 0.1%, KCl 0.1%, NaCl 1%.

Soil sampling: Soil sampling followed a randomized block design with four replicates per treatment (control + four fertilizer types). From each experimental plot, a composite sample was obtained by pooling 3–5 subsamples collected in a zigzag pattern from the 0–10 cm layer, corresponding to the active zone. Samples were properly labeled, documented, and stored under controlled conditions to preserve microbial integrity and ensure statistical reproducibility. Rhizosphere soil. At each sampling location, whole plants were excavated with surrounding soil to preserve the root system. Loose bulk soil was removed by vigorous hand-shaking for ~1 min. Roots with adhering soil were placed into sterile 50 mL polypropylene tubes containing 0.9% (*w*/*v*) NaCl (50 mL per plant) and shaken on an orbital shaker by hand for 10 min to dislodge rhizosphere particles. To recover the rhizosphere fraction, suspensions were centrifuged at 3000× *g* for 5 min and the supernatant discarded; the pellet (rhizosphere soil) was retained. A second wash with 0.9% NaCl + 0.01% (*v*/*v*) Tween-80 was performed when a more strongly adhering rhizoplan fraction was required. Visible root fragments and large debris were removed by hand; soil was homogenized and sieved through a 2 mm mesh prior to analysis.

Physico-chemical parameters: Soil moisture content was determined gravimetrically using the oven-drying method. Fresh soil samples were weighed and dried in a hot air oven at 110 °C for 24 h until constant weight was achieved. Moisture percentage was calculated based on the difference between fresh and dry weight, reflecting the soil’s water-holding capacity and its influence on microbial activity. Soil temperature was measured in situ using a calibrated bimetallic thermometer inserted at a depth of 10–20 cm, representing the biologically active soil layer where root–microbe interactions occur. The thermometer was allowed to equilibrate before readings were taken to ensure accuracy. Soil pH was determined using the colorimetric method. For each sample, 10 g of soil was suspended in an equal volume (1:1 ratio) of distilled water and allowed to settle. A pH indicator strip was immersed in the supernatant solution, and the resulting color change was compared to a standardized color chart. Soil pH is a critical factor regulating microbial community composition, enzyme stability, and nutrient availability.

Heterotrophic Culturable Bacteria (THCB): To quantify total bacterial counts and assess dehydrogenase activity, soil samples were collected from the upper 10 cm of the soil profile. Ten individual samples were collected from each experimental plot and subsequently mixed in the laboratory to achieve homogenization. Following homogenization, three aliquots of 1 g of soil each were taken from the mixed samples and inoculated in triplicate for the estimation of heterotrophic bacteria and dehydrogenase activity. The enumeration of cultivable bacteria was performed on Nutrient Agar (Oxoid beef extract 0.3 g; peptone 0.5 g, sodium chloride 0.5 g, agar 1.5 g, distilled water, 100 mL, pH = 7.2) and on A2R Agar (proteose peptone 0.5; casein hydrolysate, 0.5; yeast extract 0.5; glucose, 0.5; starch, 0.3; sodium pyruvate, 0.3; dipotassium phosphate, 0.3; magnesium sulfate, 0.024; agar, 15, pH = 7.2). Prior to inoculation, soil samples were diluted in a sterile saline solution (0.85% NaCl) and thoroughly stirred to ensure optimal homogenization. Following dilution and homogenization, 0.1 mL of each sample was inoculated by spreading on the surface of solidified media and incubated for 3–4 days at 28 °C. Subsequently, the colonies were counted, and the total bacterial population was expressed as colony-forming units per gram of soil (CFU/g soil). To ensure that only bacterial colonies were enumerated on the non-selective nutrient agar plates, colonies were differentiated based on incubation time, colony morphology, and microscopic examination. Plates were incubated at 28 ± 2 °C and observed daily for seven days. Colonies that appeared within 24–72 h, exhibited smooth, moist, or mucoid textures, and lacked aerial mycelium were classified as bacterial. In contrast, colonies that developed after 5–7 days typically showed filamentous or fuzzy growth consistent with fungal morphology. Only morphologically and microscopically confirmed bacterial colonies were included in the colony-forming unit (CFU) counts.

Estimation of Dehydrogenase Activity (DHA): The assessment of dehydrogenase activity was conducted following the protocol outlined by Małachowska-Jutsz and Matyja [13], with minor modifications to the conventional method. After homogenization, the soil samples were weighed, and each test tube received 1 g of the soil sample. Three test tubes (triplicates) were utilized for each experimental treatment. Each test tube containing 1 g of soil was supplemented with 900 µL of phosphate buffer (pH 7.4), 80 µL of 5% glucose solution, and 20 µL of 5% TTC (2,3,5-triphenyl-2H-tetrazolium chloride—Merck, Darmstadt, Germany). Additionally, for each experimental treatment, a control variant, inactivated by autoclaving, was included to differentiate between biological and chemical reduction in TTC. All experimental treatments, including controls, were assessed in triplicate, with the final results expressed as the mean value. Following the addition of TTC and phosphate buffer, incubation was conducted for 24 h at 28 °C. After incubation, the tubes were centrifuged, and the supernatant was discarded. TPF (1,3,5-triphenyl-tetrazolium-formazan) was extracted by adding 1 mL of methanol (Merck) to each test tube, and the tubes were agitated for optimal extraction. The test tubes were centrifuged again (4000 rpm for 15 min), and the supernatant containing TPF was transferred to separate test tubes. This extraction procedure was repeated 4–5 times until the solvent became colorless. The extracted TPF was quantified using a Jasco UV-Vis spectrophotometer at a wavelength of 485 nm. The concentration of TPF was determined using a standard curve with concentrations ranging from 1 to 30 µg TPF/mL.

Estimation of DHA in rhizosphere soil: For each experimental treatment, 1 g of fresh rhizosphere soil was placed into three replicate test tubes. To each tube, the following reagents were added: 900 µL of phosphate buffer (pH 7.4), 80 µL of 5% glucose solution, 20 µL of 5% TTC solution (Merck). Samples were mixed thoroughly and incubated for 24 h at 28 °C. TPF was extracted using methanol and quantified spectrophotometrically, as previously described.

Evaluation of the in vitro effect of fertilizers on DHA: Soil used for this assay was collected from unfertilized control plots, following the same protocol previously described for sampling. After collection, the soil samples were thoroughly homogenized to ensure uniformity. For each treatment, reaction mixtures were prepared in test tubes by adding 1 g of air-dried, sieved soil, 800 μL of phosphate buffer (pH 7.4) to maintain optimal enzymatic conditions, 100 μL of a 10% fertilizer solution, 80 μL of a 5% glucose, and 10 μL of a 5% 2,3,5-triphenyltetrazolium chloride (TTC) solution Control treatments received no fertilizer addition. All assays were performed in triplicate to ensure reproducibility and statistical reliability.

Statistical analysis: All data were analyzed using one-way analysis of variance (ANOVA) to determine whether significant differences (*p* < 0.05) existed between treatments (control vs. fertilized variants) for both total heterotrophic bacterial counts and DHA levels. Statistical analyses were performed using one-way analysis of variance (ANOVA) to evaluate differences among fertilizer treatments. This method was chosen because the experimental design included a single factor (fertilizer type) with multiple treatment levels, and ANOVA is appropriate for assessing whether mean differences among groups are statistically significant. When ANOVA indicated significance, Tukey’s Honestly Significant Difference (HSD) test was applied for pairwise comparisons of heterotroph counts, while the Games–Howell post hoc test was employed for DHA comparisons due to potential variance heterogeneity. Statistical evaluations were performed using the STW Statistics 18 software package.

## 3. Results

### 3.1. Soil Physico-Chemical Parameters

**Figure 1:**
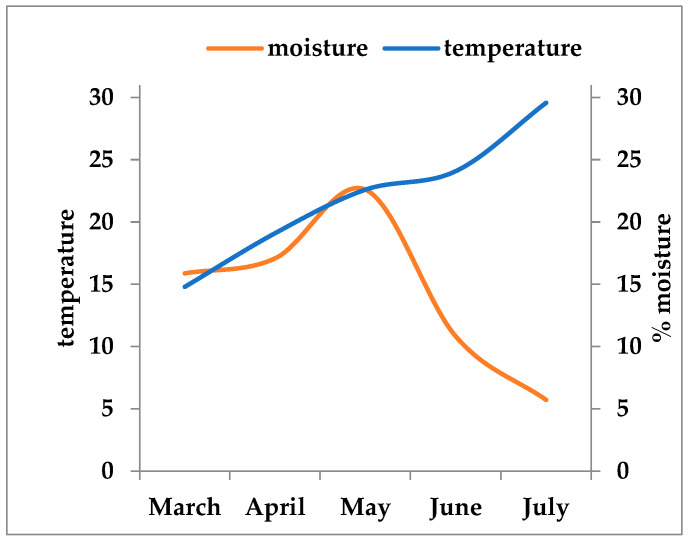
Seasonal dynamics of soil temperature, moisture, and pH during the study period (March–July). Temperature (°C) and moisture (%) showed contrasting trends, with increasing temperatures coinciding with a continuous decline in soil moisture. Soil pH exhibited only minor fluctuations, remaining between 6.1 and 6.7 throughout the experimental period.

During the experimental period, soil temperature exhibited substantial seasonal variability, ranging from 14.8 °C in March to 29.6 °C in July. Moisture showed two opposite trends (Figure 1). It initially increased from 15.88% (March) to 22.6% (May). In the second part of the observation period moisture decreased in June (10.83%) and reached its minimum value in July (5.72%) which coincided with the maximum recorded temperature (29.58 °C). In contrast, soil pH showed only minor, insignificant fluctuations, remaining within a slightly acidic to near-neutral range (6.1–6.7) (Figure 1).

### 3.2. Dynamics of Total Heterotrophic Culturable Bacteria (THCB)

**Figure 2:**
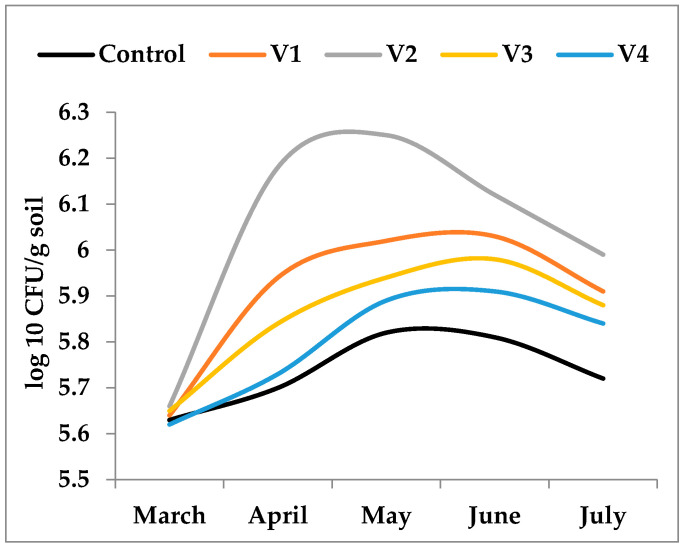
Density of THCB (log_10_ CFU g^−1^ soil) in unfertilized control (C) and fertilized treatments (V1, V2, V3, V4) from March to July. Fertilizer type strongly influenced both the magnitude and temporal stability of bacterial populations. Treatments with labile organic inputs (V2—Figure 3) induced rapid increases followed by declines, while recalcitrant organic amendments (V1, V3) promoted sustained but moderate enrichment. Mineral fertilization (V4) supported comparatively lower bacterial densities, likely linked to plant root exudation.

At the onset of this study (March), the density of cultivable heterotrophic bacteria showed little variability among control and fertilized treatments (Figure 2). Following fertilizer application, bacterial populations increased markedly during the first two months, with distinct trajectories depending on the fertilization regime. Across the entire study period, the highest mean bacterial densities were recorded in V1 (6.19 log_10_ CFU g^−1^ soil) and V3 (6.10 log_10_ CFU g^−1^ soil). The overall maximum was reached in May in the Bio Ostara treatment (V2), with 6.39 log_10_ CFU g^−1^ soil. In the other treatments, peak densities were observed in June and ranged from 6.12 log_10_ CFU g^−1^ soil (V4) to 6.24 log_10_ CFU g^−1^ soil (V1). In the unfertilized control, the maximum population reached only 5.98 log10 CFU g^−1^ soil in June.

These results indicate that the magnitude and persistence of bacterial growth were strongly determined by the chemical nature and degradability of the applied substrates. Easily mineralizable organic inputs generated rapid increases in bacterial density but were followed by pronounced temporal fluctuations, while more recalcitrant organic amendments sustained a gradual and more stable enrichment of the heterotrophic population.

**Figure 3:**
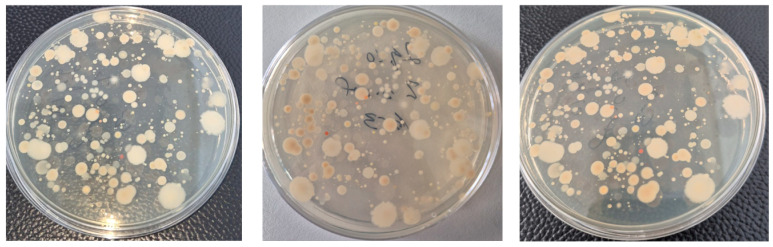
Colonial morphology of heterotrophic bacteria after inoculation of soil samples on Nutrient Agar and A2R Agar (variant V2).

For THCB, the one-way ANOVA indicated statistically significant differences in mean levels across the five groups (F(4, 20) = 6.368, *p* = 0.003). Post hoc analysis using Tukey’s Honestly Significant Difference (HSD) test revealed that the Control group had significantly lower mean density compared to both the V1 and V3 groups (*p* < 0.05).

### 3.3. Dehydrogenase Activity (DHA)

**Figure 4:**
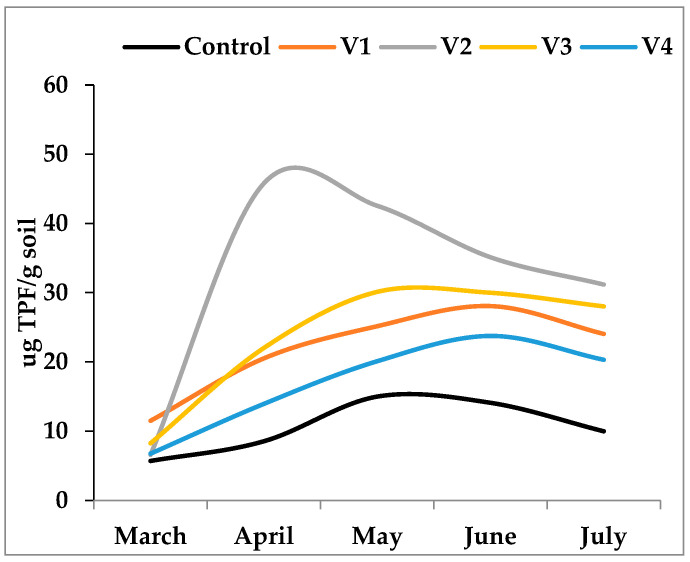
Dehydrogenase activity (μg TPF g^−1^ soil) in control (C) and fertilized treatments (V1, V2, V3, V4) over the experimental period. Peaks in activity varied according to the degradability of organic substrates: rapid and early maximum in V2 (April), delayed and moderate maxima in V1 and V3 (June), and relatively low activity in V4, closely resembling the control.

Patterns of microbial oxidative activity, as indicated by dehydrogenase (DH) assays, revealed clear differences among treatments (Figure 4). On average, DH activity was highest in V2 (25.44 μg TPF g^−1^ soil) and lowest in V4 (13.89 μg TPF g^−1^ soil). Overall, these results demonstrate that the nature of the fertilizer strongly modulates both the growth of culturable heterotrophic bacteria and their associated metabolism. Organic amendments with easily degradable carbon fractions stimulate rapid but transient microbial activity, whereas more complex organic inputs maintain moderate but longer-lasting activity. Mineral fertilization, in contrast, sustains microbial activity indirectly through plant-mediated organic matter release.

**Figure 5:**
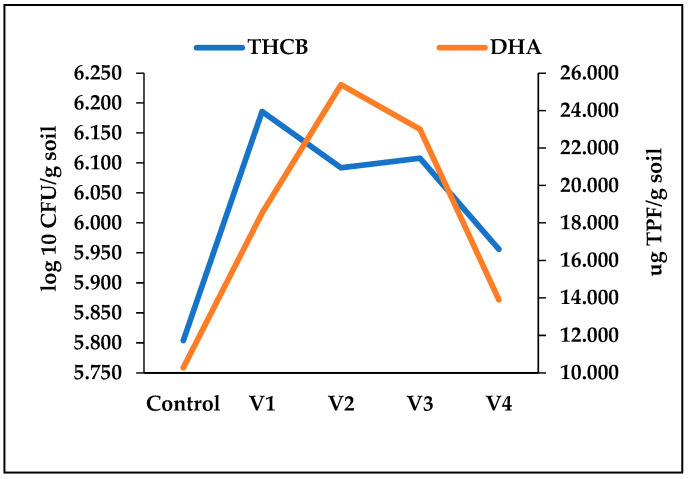
Relationship between Average THCB Density and DHA in Soil. The positive correlation (r = 0.79) observed between average bacterial density and average dehydrogenase activity illustrates a fundamental principle in soil microbiology: the close link between microbial biomass and enzymatic function. This relationship demonstrates the tendency of these two parameters to fluctuate in tandem, highlighting the crucial role that active, metabolically diverse bacterial populations play in the decomposition of organic matter.

For DHA, the ANOVA also indicated statistically significant differences (F(4, 20) = 5.642, *p* = 0.004). The Games–Howell post hoc analysis for DHA reveals several statistically significant differences: (i) the mean DHA level of the Control group (M = 12.385) was significantly lower than that of V1 (M = 27.428), V2 (M = 32.238), and V3 (M = 27.923) (all *p* < 0.001); (ii) the mean DHA level of V4 (M = 17.772) was significantly lower than that of V1 (M = 27.428) (*p* = 0.038), V2 (M = 32.238) (*p* = 0.002), and V3 (M = 27.923) (*p* = 0.026). Dehydrogenase activity, a key indicator of overall microbial metabolic activity, is directly proportional to the rate at which microorganisms process substrates. The significant positive relationship (Figure 5) suggests that as bacterial populations increase, their collective enzymatic output—specifically the reduction in substrates catalyzed by dehydrogenases—also rises. This indicates that a greater number of active, cultivable bacteria are capable of processing a large quantity of complex organic compounds efficiently and rapidly, contributing significantly to nutrient cycling and overall soil health.

### 3.4. DHA in Rhizosphere

**Figure 6:**
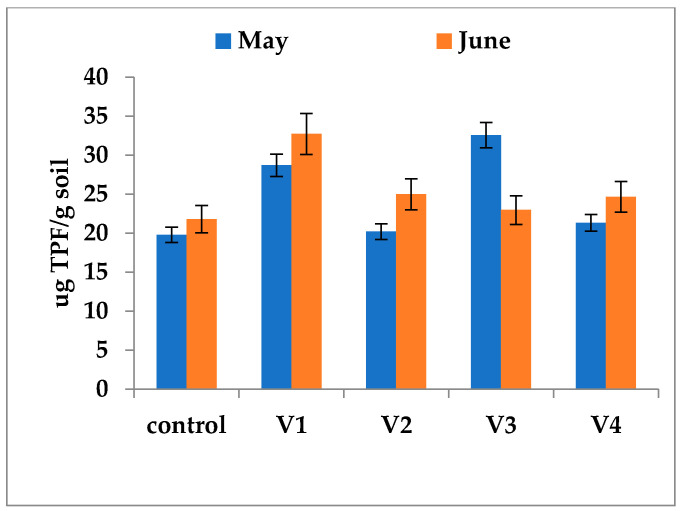
In vitro DH activity in soil samples collected from the rhizosphere of rapeseed plants in the experimental plots. Soil samples were collected from the fertilized and control variants in May and June, a period encompassing the flowering and early fruiting stages, when microbial metabolic activity is most vigorous. The distribution pattern of DHA between bulk soil and rhizosphere was different and statistically significant (*p* < 0.05). Error bars represent standard deviations of replicate measurements.

Overall, at the time of sampling (May and June), dehydrogenase activity (DHA) was generally elevated in the rhizosphere compared to the bulk soil of the corresponding experimental plots, with the exception of variant V2. The most pronounced DHA levels were measured in variants V1 (32.72 µg TPF/g soil in June) and V3 (32.55 µg TPF/g soil in May). In contrast, variants V2 and V4 exhibited intermediate activity, with values ranging from 20.2 µg TPF/g soil (V2 in May) to 24.66 µg TPF/g soil (V4 in June) (Figure 5). In the rhizosphere of plants from the unfertilized control treatment, DHA recorded a somewhat reduced value, ranging from 19.78 to 21.78 µg TPF/g soil (Figure 6). Nevertheless, this activity was still greater than the DHA level found in the corresponding nonfertilized control soil (Figure 6). The spatial profile of DHA appears to be, at least partially, independent of fertilizer use and seems to be governed by plant metabolic processes rather than the specific composition of the soil amendments.

### 3.5. The In Vitro Influence of Fertilizers on Bacterial DHA

**Figure 7:**
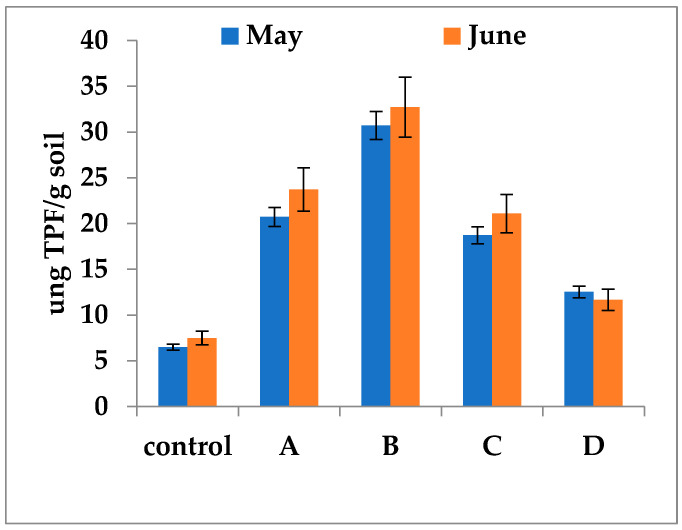
Estimation of the direct in vitro influence of fertilizers on DHA. A—Fertil 4-5-7; B—Bio Ostara N; C—P35 Bio; D—Bioaktiv. The samples were collected in two different months, May and June from the unfertilized control plots. The experiment aimed to highlight the effect of fertilizers on DHA independently of the influence of physicochemical (temperature, pH, humidity, adsorption, precipitation, volatilization) and biological (higher organisms) factors in the natural soil in which case the entire amount of fertilizers is available to bacterial metabolism. The one-way ANOVA indicated statistically significant differences in mean levels across the five groups. Post hoc analysis using HSD test revealed that the Control group had significantly lower mean density compared to both the A, B and C groups (*p* < 0.05). Error bars represent standard deviations of replicate measurements.

To evaluate the direct influence of fertilizers, samples from unfertilized control plots were supplemented with the respective fertilizers, and DHA was measured according to the previously described protocol. Laboratory experiments indicated that fertilizers exerted a differentiated direct effect on DHA (Figure 7).

The greatest increase was observed in variant B, with values ranging from 30.72 to 32.72 µg TPF/g soil. Variants A and C showed intermediate activities (18.72–21.08 µg TPF/g soil). The lowest values (11.65–12.52 µg TPF/g soil) were detected in variant D treated with mineral fertilizers, though these values remained higher than those in the control (Figure 7). These laboratory experiments, carried out under uniform physicochemical conditions and in the absence of higher organisms, confirmed a trend consistent with that observed under field conditions.

## 4. Discussion

**Figure 8:**
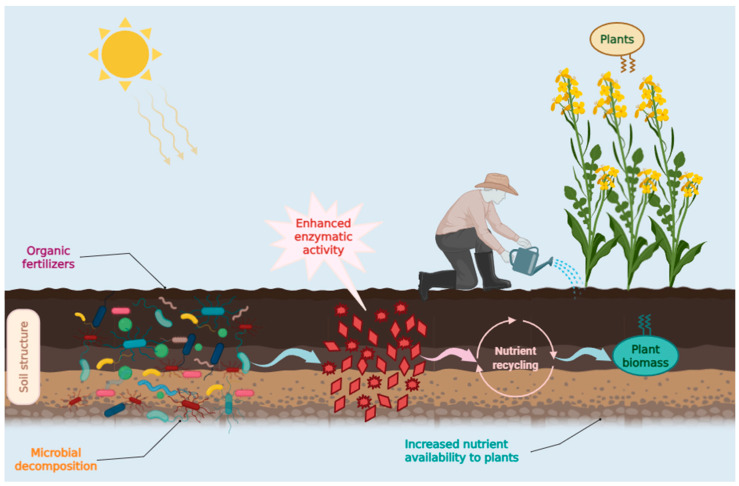
The Role of Microorganisms and Their Enzymatic Functions in the Decomposition, Nutrient Recycling, and Biotransformation of Organic Fertilizers into Plant-Available Inorganic Forms.

As discussed in the Introduction, the influence of the fertilizers in question was evaluated by measuring changes in the density of cultivable bacteria and in dehydrogenase activity (DHA). Although the cultivable fraction of soil microorganisms represents only a small portion of total bacterial diversity, its ecological and agronomic importance is disproportionately high. The bacteria recoverable through cultivation generally correspond to copiotrophic *r*-strategists (e.g., *Pseudomonas*, *Bacillus*) characterized by rapid growth kinetics and an ability to exploit readily available organic substrates [27,28]. These taxa are the primary first responders following fertilizer application, driving the short-term decomposition of labile organic matter and accelerating nutrient mineralization. In contrast, molecular surveys provide a broader overview of community structure, but they often emphasize slower-growing oligotrophic groups [27,29,30]. Moreover, many of the most effective plant growth–promoting rhizobacteria and phosphate-solubilizing strains used in agricultural bioinoculants were originally identified through cultivation-dependent approaches, underscoring the applied value of this microbial fraction. Similarly, dehydrogenase activity represents a robust functional proxy for microbial metabolism, as it reflects the intracellular redox activity of viable microbial cells. Unlike extracellular enzymes, which can persist in soil after cell death, dehydrogenases are tightly associated with living biomass [31,32] and therefore provide a sensitive indicator of shifts in microbial activity under different fertilization regimes [33]. Taken together, the assessment of cultivable heterotrophs and dehydrogenase activity captures the functionally active, fast-growing microbial fraction most directly responsible for short-term nutrient cycling and plant nutrient availability—information that is complementary to, but not replaceable by, molecular community profiling. In soils amended with organic fertilizers enriched in easily degradable organic matter, fast-growing heterotrophs play a pivotal role in the initial stages of nutrient cycling. By rapidly metabolizing carbon- and nitrogen-rich inputs, they stimulate early plant growth responses and contribute to short-term increases in soil fertility [34,35,36]. Furthermore, their metabolic by-products—such as organic acids, extracellular enzymes, and low-molecular-weight signaling compounds—exert a stimulatory effect on the activity and succession of slower-growing microbial guilds (*K*-strategists), thereby shaping the functional trajectory of the soil microbiome [37,38,39]. Although culturable heterotrophs represent only a limited fraction of the overall bacterial diversity in soils, their ecological significance lies in their ability to temporarily dominate microbial niches due to competitive growth advantages [40,41]. Empirical evidence demonstrates that compost and organic amendments can induce rapid surges in culturable heterotrophs, paralleled by increases in enzyme activities such as dehydrogenase, phosphatase, and β-glucosidase [22,23,35,36]. Thus, evaluating the dynamics of these rapidly proliferating taxa provides critical insights into the short-term microbial and agronomic consequences of organic fertilizer applications, complementing broader assessments of long-term soil health and microbial diversity [38,39,40,41]. Organic fertilizer V2 stimulated the most rapid initial growth, but bacterial density subsequently declined sharply from late April onward. Organic fertilizers V1 and V3 induced a more moderate but sustained increase, consistent with the slower degradation of their organic fractions and the gradual release of nutrients. Mineral fertilizer V4 supported comparatively lower bacterial densities, with increases likely attributable to the release of root-derived organic substrates rather than direct fertilization effects. ANOVA indicated a statistically significant difference among the group means (*p* = 0.003) of experimental variants. Subsequent pairwise comparisons identified that the control group had significantly lower mean THCB density than both V1 and V3. The temporal dynamics of maximum activity reflected substrate accessibility: V2 (rapidly degradable organic matter) exhibited the shortest response time, reaching its peak in April with 45.75 μg TPF g^−1^ soil. V1 and V3 (more recalcitrant organic matter) achieved later maxima in June, with comparatively lower values of 24.1 and 30.5 μg TPF g^−1^ soil, respectively. V4 (mineral fertilizer) showed a peak in May (17.22 μg TPF g^−1^ soil), close to the control (15.22 μg TPF g^−1^ soil), and both displayed similar temporal patterns. This suggests that, under mineral fertilization, microbial oxidative activity was primarily sustained by root-derived organic inputs rather than the fertilizer itself. The variability of bacterial communities is significantly influenced by seasonal changes in temperate regions [20]. In our experiments, THCB density increased with increasing temperature until the end of May, after which the density stabilized and then decreased slightly towards the end of the observation period (July) mainly due to the drastic decrease in moisture. This indicated a weak or mixed relationship between THCB density and temperature due to the interference between the two essential factors with contrasting evolution during the analyzed period. A similar trend was also noted for DHA, which was characterized by a significant increase until the end of May followed by a slight decrease towards the end of the observation period. The two parameters, THCB and DHA, demonstrated a close positive correlation (r = 0.79) and statistically significant (*p* < 0.001) demonstrating the dependence of microbial metabolic activities on the presence of culturable bacteria. The rhizosphere is the narrow, dynamic zone of soil immediately surrounding plant roots that is profoundly influenced by the root’s secretions, known as root exudates, and the associated soil microorganisms. It is a “hotspot” for microbial activity, with a significantly higher density and diversity of microbes compared to the bulk soil. The rhizosphere is a complex micro-ecosystem where plants actively shape their microbial partners to enhance their own growth, acquire essential nutrients, and protect themselves from disease, thereby playing a fundamental role in soil fertility and ecosystem function [42,43,44]. In our experiments, DHA in the rhizosphere showed higher levels on average than DHA in the rootless soil, but the differences were not statistically significant. The composition of the fertilizers had a pronounced effect on enzymatic activity. Dehydrogenase activity varies depending on factors such as soil type [45], vegetation cover [9,46,47], the presence of pollutants (e.g., herbicides), and land management practices (e.g., intercropping) [48]. Organic amendments consistently yielded higher levels of dehydrogenase activity compared to their inorganic counterparts [49]. It has been well-established that the appropriate structure and optimal functioning of microbial communities are essential for the productivity of agroecosystems [50]. Both microbial density and the activity of microbial communities serve as effective indicators of soil quality [51]. Significant differences in soil microbial composition and activity were observed following fertilization [52,53,54]. The combined application of organic and inorganic fertilizers markedly enhanced bacterial diversity [20,55]. Organic fertilizers have been shown to exert a positive influence on soil microbial biomass [56,57]. Moreover, microbial diversity was greater in plots treated with a combination of chemical and organic fertilizers compared to plots treated solely with chemical fertilizers [58,59]. Additionally, a substantial increase in enzymatic activity was recorded following the incorporation of organic matter [60]. Conversely, the application of inorganic fertilizers led to a reduction in both microbial density and diversity [61]. Based on average values, the most significant impact on the increase in the density of culturable heterotrophic bacteria was observed with the Bio Ostara N fertilizer (V2), notable for its high organic nitrogen content (12%). The second highest bacterial density was found in samples from the V1 variant, which received Fertil 4-5-7 fertilizer, containing a total organic matter content of 48%. A relatively high bacterial density was also noted in the V3 plot, fertilized with P35 Bio, which has a high phosphorus content (28%). The V4 variant, treated with BioAktiv featuring a mineral composition, displayed a slightly lower yet consistently higher bacterial density compared to the control group. Fertilization can influence the activity of microbial dehydrogenases, which serve as indicators of soil biological activity [62,63,64]. Most studies have indicated that inorganic fertilizers exert a lesser impact on dehydrogenase activity compared to organic fertilizers [65,66,67]. Significant variations in dehydrogenase activity have also been noted between conventional and organic agricultural practices. Conventional farming methods that employ inorganic fertilizers and pesticides tend to exhibit lower dehydrogenase activity [68]. An increase in organic input is, in most instances, correlated with an elevation in soil dehydrogenase activity [69]. Moreover, a 20% reduction in the application of inorganic fertilizers did not significantly influence soil dehydrogenase activity [70]. The activity of microbial dehydrogenases was also found to be greater in soils treated with organic fertilizers [71]. Additionally, soil type has a significant effect on the activity of microbial dehydrogenases [49]. Dehydrogenase activity is further influenced by the depth of the soil profile [72]. Organic farming practices stimulate the growth of microbial biomass by 32% to 84% and enhance overall enzymatic activity, including dehydrogenases [73]. The combination of organic and inorganic fertilizers on agricultural land has been shown to increase microbial dehydrogenase activity [74]. A similar trend observed in our experiment for bacterial density was noted in the dehydrogenase activity, with the highest intensity recorded in variant V2, followed closely by variants V3 (Figure 4). Variant V4, treated with P 35 Bio, exhibited a somewhat lower effect on dehydrogenase activity, although it was still higher than the enzymatic activity observed in the control plot (V1) (Figure 4). It is likely that variant V3, despite containing organic matter, demonstrates reduced dehydrogenase activity due to the lower bioavailability of its organic content, which may be more challenging to degrade. This suggests that fertilizers rich in organic matter, particularly those with high concentrations of organic nitrogen, promote the growth of heterotrophic microbial communities and enhance their enzymatic activity. Overall, our findings align with previous studies regarding the impact of organic and inorganic fertilizers on soil microbial communities and their activities (Figure 7). Soil moisture content significantly influences both the density of microbial communities and the intensity of carbon and nitrogen cycling [75]. Different microbial groups have optimal moisture levels that vary between 20% and 60%, while the optimal moisture content for enzyme activity, including that of dehydrogenases, is generally around 20% [76]. Tomar and Baishya [77] demonstrated that moisture is a key factor regulating microbial biomass and dehydrogenase activity in semi-arid climate conditions. They reported that temperature and the seasonal vegetation cycle profoundly influence soil microbial processes, including dehydrogenase activity. Research indicates that fluctuations in moisture affect microbial enzyme activity in soils [78]. The most pronounced sensitivity, in comparison to other enzymes, was observed for dehydrogenases [78]. Moreover, moisture was positively correlated with dehydrogenase activity [79]. Luo et al. [32] found that organic manure positively affects microbial biomass and dehydrogenase activity, indicating that the type of fertilizer alters soil properties and fertility status. The seasonal variation in dehydrogenase activity depends on the specific soil horizon and organic matter concentration. The authors revealed that while dehydrogenase activity correlates with organic matter concentration, in certain contexts (such as pine forests), it does not exhibit significant seasonal fluctuations [80]. Generally, dehydrogenase activity has been shown to positively correlate with soil pH, calcium (Ca), magnesium (Mg), potassium (K), and moisture content in forest soils [81]. Land management practices (Figure 8) that affect the organic matter content of the soil play a crucial role in controlling microbial biomass and enzymatic activity [82]. Dehydrogenase activity is influenced by the tillage system, with higher activity observed in no-tillage soils [83]. In studies conducted on coal heaps, Kompała-Bąba [84] noted that soil pH, along with moisture significantly impacts the dynamics of microbial communities and dehydrogenase activity. The factors mentioned above and their variability illustrate the complex relationship between microbial density, enzymatic activity, and the organic and inorganic matter added to the soil as fertilizers. The results of this study demonstrated that organic fertilizer application significantly increased both total heterotrophic bacterial (THCB) density and dehydrogenase (DHA) activity compared with inorganic fertilization. This suggests that the type and quality of fertilizer inputs play a critical role in shaping microbial community dynamics and enzymatic functioning in soil ecosystems. While the general influence of organic matter on microbial activity is well recognized, understanding why organic inputs elicit a stronger microbial response requires consideration of their biochemical characteristics and effects on soil properties. The greater bacterial density and dehydrogenase activity observed under organic fertilizer application can be mechanistically explained by the biochemical composition of the added organic matter. Organic fertilizers typically contain easily degradable substrates such as simple sugars, amino acids, and organic acids, which are rapidly metabolized by heterotrophic microorganisms. The catabolism of these labile carbon sources enhances microbial respiration and generates intracellular reducing equivalents (NADH and FADH_2_), which fuel the soil electron transport system and stimulate dehydrogenase activity (Figure 8).

Elevated dehydrogenase activity, in turn, reflects increased microbial oxidative metabolism and overall biochemical turnover in the soil. In contrast, inorganic fertilizers provide mineral nutrients but lack organic carbon inputs, limiting direct stimulation of microbial metabolism. Additionally, the incorporation of organic matter improves key soil physical and chemical properties, including aggregation, porosity, and moisture-holding capacity, which create more favorable microenvironments for microbial proliferation and enzyme stability. These microbial and physicochemical interactions not only enhance THCB density and DHA but also contribute to improved nutrient mineralization and the buildup of soil organic carbon—important indicators of long-term soil fertility and biological quality. Overall, these findings highlight that the stimulation of microbial and enzymatic activity by organic fertilizers is not merely a short-term biological response, but part of a broader process that enhances nutrient cycling efficiency and soil health over time. Future studies incorporating detailed carbon fractionation and microbial functional profiling could further clarify these linkages and support the development of more sustainable fertilization strategies.

## 5. Conclusions

Organic amendments stimulated microbial growth and enzymatic activity more effectively than inorganic fertilizers, underscoring the importance of organic nutrient sources for maintaining soil biological fertility. Fertilizers rich in readily mineralizable organic nitrogen induced the greatest increases in bacterial density and dehydrogenase activity, reflecting a higher degree of substrate accessibility and microbial utilization. Temporal discrepancies between DHA and bacterial population peaks indicate that microbial enzymatic responses precede biomass proliferation, emphasizing DHA as a sensitive early indicator of soil biological activity. The quality and biochemical composition of organic inputs were decisive in shaping the magnitude and persistence of microbial responses, highlighting the role of substrate lability in regulating soil microbial dynamics. Overall, organic fertilization enhances soil microbiological function, contributing to improved soil health and potential long-term sustainability of agroecosystems compared with inorganic fertilization.

## Data Availability

All data generated or analyzed during this study are included in this published article.
